# *Cryptocarya alba* and *Laureliopsis philippiana* Essential Oil-Loaded Hydrogels with Antibacterial Activity Against *Staphylococcus pseudintermedius*: Potential Topical Candidates for Canine Pyoderma

**DOI:** 10.3390/vetsci13060544

**Published:** 2026-05-31

**Authors:** Martina Jacobs, Noelia Valdivia, Martín Varas, Paola Ramos, Flavia Bruna, Gabriela Valenzuela-Barra, Olosmira Correa, Antonia Díaz, Gabriela Maturana, Irene Martínez, Francisco Abusleme, Belén Rivera, María Olga Bargsted, Daniela Siel, Jessica Bravo

**Affiliations:** 1 Laboratory of Bioactive Natural Products, School of Medicine, Center for Biomedical Research, University Diego Portales, Ejército 141, Santiago 8370007, Chile; martinajacobs@ug.uchile.cl (M.J.); noelia.valdivia_c@mail.udp.cl (N.V.); martin.varas@ug.uchile.cl (M.V.); proyectos.pramos@gmail.com (P.R.); flabruna@gmail.com (F.B.); gabriela.m.valenzuela@ciq.uchile.cl (G.V.-B.); ocorrea@ciq.uchile.cl (O.C.); antonia.diazp@mayor.cl (A.D.); gabriela.maturana.alvarez@gmail.com (G.M.); 2 Department of Chemical Engineering, Biotechnology and Materials, Faculty of Physical and Mathematical Sciences, University of Chile, Beauchef 851, Santiago 8370458, Chile; imartinez@ing.uchile.cl; 3 Hormone and Cancer Biology Laboratory, Institute of Experimental Medicine and Biology of Cuyo (IMBECU), CONICET CCT Mendoza—National University of Cuyo (UNCuyo), Mendoza 5500, Argentina; 4 Center of Odontological Research (CIO) from the Faculty of Odontology, National University of Cuyo (UNCuyo), Mendoza 5500, Argentina; 5 Department of Pharmacological Chemistry and Toxicology, Faculty of Chemical and Pharmaceutical Sciences, University of Chile, Carlos Lorca Tobar 964, Santiago 8380492, Chile; 6 School of Biotechnology, Faculty of Sciences, Engineering and Technology, Mayor University, Camino La Pirámide 5750, Santiago 8580000, Chile; 7 School of Veterinary Medicine, Faculty of Medicine and Health Sciences, Mayor University, Camino La Pirámide 5750, Santiago 8580000, Chile; fabusleme@gmail.com (F.A.); mobargsted@gmail.com (M.O.B.); 8 Oftaderm Veterinary Clinic, Av. Francisco Bilbao 7341, La Reina, Santiago 7850003, Chile; belen.rivera@oftaderm.cl; 9 Center for Biomedicine, Mayor University, Camino La Pirámide 5750, Santiago 8580000, Chile

**Keywords:** canine pyoderma, *Staphylococcus pseudintermedius*, bioactive hydrogel, essential oil, antibacterial activity, *Laureliopsis philippiana*, *Cryptocarya alba*, natural products

## Abstract

Background/Objectives: The increasing emergence of antimicrobial-resistant *Staphylococcus pseudintermedius* has limited the effectiveness of conventional therapies for canine pyoderma, highlighting the need for alternative topical strategies. This study aimed to develop hydrogels incorporating essential oils (EOs) from Peumo (*Cryptocarya alba*) and Tepa (*Laureliopsis philippiana*) as potential topical treatments against *Staphylococcus pseudintermedius* skin infections in veterinary medicine. Methods: EOs were obtained by steam distillation, chemically characterized by gas chromatography–mass spectrometry (GC–MS), and evaluated for antibacterial activity against *S. pseudintermedius* strains. Carbopol^®^-based hydrogels incorporating the EOs of *C. alba* (HCA), *L. philippiana* (HLP), and a control vehicle (HVE) were formulated and characterized in terms of physicochemical properties, microbiological safety, and stability under accelerated and refrigerated conditions. Preclinical dermal safety was evaluated in BALB/c mice by repeated topical administration for five days. The analysis included clinical observation, skin irritation scoring, and histological analysis. Additionally, a preliminary microbiological evaluation was conducted in client-owned dogs with superficial pyoderma to assess the performance of the formulations in the target species. Skin lesion swabs were collected at baseline and after 21 days of treatment, followed by bacterial culture and automated identification using the VITEK^®^ system. Bacterial detection and bacterial load were evaluated to determine changes in microbiological status over the treatment period. Results: GC–MS analysis identified sabinene and eucalyptol as the main compounds in *CA-EO*, and linalool, eucalyptol, and safrole in *L. philippiana* EO. Both EOs exhibited moderate antibacterial activity against *S. pseudintermedius* (inhibition zones 4.9–10.8 mm; MIC ≥ 2.048 mg mL^−1^). The hydrogels were microbiologically safe. Among formulations, HLP demonstrated superior physical stability and comparable rheological properties to the vehicle. In vivo safety evaluation demonstrated no signs of systemic toxicity, behavioral alterations, or skin irritation, and histological analysis confirmed preserved skin architecture without evidence of inflammation or tissue damage. In the preliminary microbiological evaluation in dogs, all animals were positive for *Staphylococcus* spp. at baseline. On Day 21, bacterial elimination was observed in the active treatment groups, but not in the HVE group, with elimination rates of 50.0% for Inveclor^®^ and 25.0% for both HCA and HLP. In parallel, HLP showed the highest proportion of dogs reaching minimal bacterial load levels (75%), followed by Inveclor^®^ (50.0%) and HCA (37.5%), whereas no dogs in the vehicle group reached this category. Conclusions: EOs from *C. alba* and *L. philippiana* presented antibacterial activity and were successfully incorporated into microbiologically safe hydrogel formulations. Notably, HLP demonstrated superior stability and a favorable preclinical safety profile, supporting its potential. In the preliminary microbiological evaluation in dogs, numerical differences in bacterial elimination and bacterial load categories were observed among groups; however, these differences were not statistically significant and should be interpreted as exploratory.

## Simple Summary

Conventional management of bacterial skin infections in dogs relies on topical antiseptics and systemic antibiotics, which may cause adverse reactions and contribute to antimicrobial resistance. This study introduces novel hydrogel formulations incorporating essential oils (EOs) from *Cryptocarya alba* and *Laureliopsis philippiana* for the treatment of canine pyoderma caused by *Staphylococcus pseudintermedius*. The chemical composition of the EOs was characterized using gas chromatography–mass spectrometry, and their antibacterial activity was assessed against 12 *S. pseudintermedius* strains. The stability of the formulations and their dermal safety were evaluated, including preclinical testing in mice and preliminary clinical assessment in dogs. Both EOs showed antibacterial activity similar to that reported for other plant-derived EOs and consistent across strains. The *C. alba* EO hydrogel showed loss of consistency and phase separation after six months, while the *L. philippiana* EO hydrogel remained stable, similar to the vehicle. Both formulations were non-irritating. In dogs, the *L. philippiana* EO hydrogel showed the most favorable microbiological profile, supporting its potential as a topical treatment for canine pyoderma.

## 1. Introduction

Bacterial skin infections, particularly pyoderma, is one of the most frequent dermatological conditions in small animal practice, accounting for up to 20–30% of dermatology consultations [1,2]. In dogs, the primary etiological agent for these infections is *Staphylococcus pseudintermedius* (SP) [2,3], a common commensal of the canine skin microbiota. The conventional treatment for these infections typically involves administering systemic antibiotics [4]. However, this approach is increasingly complicated by the emergence of multidrug-resistant strains, most notably methicillin-resistant strains (MRS) [4], which pose a serious threat to the effectiveness of antimicrobial therapy. This growing crisis calls for the development of novel, site-specific treatment strategies to mitigate bacterial load at the site of infection while minimizing the widespread use of systemic antibiotics. To address this problem, hydrogels, a class of biocompatible polymeric materials with high water content, are emerging as a promising solution due to their ability to deliver therapeutic agents directly to the wound bed, coupled with their capacity to maintain a moist healing environment, presenting a compelling alternative to traditional treatments for canine pyoderma [5]. 

Natural products, particularly plant-derived essential oils (EOs) rich in terpenes and phenylpropanoids, have attracted increasing attention as potential alternatives or adjuncts to conventional antimicrobials. EOs are complex mixtures composed predominantly of terpenes, terpenoids, and phenylpropenes, which are widely recognized as the principal contributors to their biological and antibacterial activities [6,7,8]. EO constituents such as α-terpineol, linalool, eucalyptol, and α-pinene have been reported to exhibit antibacterial activity against Gram-positive and Gram-negative bacteria, including *Escherichia coli* and *Staphylococcus aureus* [9]. Other notable antibacterial components found in EOs include 1,8-cineole, linalyl acetate, and pulegone [10,11]. EOs possess a unique dual potential in dermatology, acting both as antimicrobial agents and as restorative compounds promoting overall skin health and wound management [12]. EOs contain bioactive components that support the healing process due to their notable anti-inflammatory activity and antioxidant properties, which help protect cells from oxidative stress and enhance tissue regeneration [13]. Specialized formulations, such as hydrogels loaded with thyme EO, have been shown in vivo to significantly promote wound contraction and positively modulate key markers related to inflammation and tissue repair [5]. This multifunctional capacity allows EOs to address infection while simultaneously supporting the skin’s natural healing mechanisms [12].

Peumo (*Cryptocarya alba*) and Tepa (*Laureliopsis philippiana*) have been used by the indigenous communities in Chile to treat a wide range of ailments. For example, *C. alba* bark infusion has traditionally been used to treat liver disorders, while preparations combining bark and leaves have been used for rheumatism and wound healing [14]. The leaves, flowers, and bark of *L. philippiana* have been used to treat colds and skin affections [15]. Studies on the chemical composition of these plants have stated that their EOs are rich in bioactive compounds such as eucalyptol (1,8-cineole), safrole, and linalool, among other terpenes [15,16]. Despite the growing interest in EO-based formulations, there are currently no reports of hydrogel systems incorporating *C. alba* or *L. philippiana* for veterinary applications.

This study aims to develop and characterize hydrogel formulations incorporating EOs from *C. alba* and *L. philippiana* for potential veterinary application in canine pyoderma. As these EOs are being incorporated into a hydrogel pharmaceutical formulation for the first time, it is of utmost importance to assess their quality, safety, and stability. To accomplish this, the EOs’ chemical composition and antibacterial activity against SP strains were studied, and then, the formulations’ stability and microbiological safety were assessed.

## 2. Materials and Methods

### 2.1. Plant Material

Fresh leaves of *C. alba* were collected during spring (November 2024) at the end of the flowering period and before fruit formation, in Chicauma, Lampa, Metropolitan Region, Chile (33°12′3.8808″ S, 70°58′8.1156″ W). Similarly, fresh leaves of *L. philippiana* were collected in October 2024 during the anthesis period in Los Pellines, Valdivia, Los Ríos Region, Chile (39°44′42.0″ S, 73°21′25.2″ W). Voucher specimens were prepared for both species. *C. alba* (SQF 22901) was deposited in the SQF Herbarium of the Faculty of Chemical and Pharmaceutical Sciences, University of Chile, while *L. philippiana* (VALD 3560) was deposited in the Herbarium of the Universidad Austral de Chile. Plant material was processed for extraction on the day of collection or within 24 h thereafter.

### 2.2. EO Extraction

EOs extraction from both species was carried out immediately after collecting using a 10 L Clevenger-type apparatus by the steam distillation method. The process was conducted in a single batch until the entire plant material was distilled (1 h per extraction in both cases). The resulting heterogeneous mixture was then separated using a separatory funnel to recover the EO. The hydrosol was discarded. Thus, two EO were obtained: *C. alba* EO (CA-EO) and *L. philippiana* EO (LP-EO).

### 2.3. Chemical Characterization 

The chemical characterization of EOs was performed by diluting the oils in dichloromethane. A 1 μL sample was then analyzed using a gas chromatography–mass spectrometry (GC-MS) system (Thermo Scientific (Waltham, MA, USA); GC: Trace 1300, MS: TSQ 8000 Evo). The ionization was carried out by electron ionization at 70 eV. The injector was set at 250 °C in split less mode. The transfer line temperature was maintained at 250 °C. Helium was used as the carrier gas at a flow rate of 1.2 mL/min, and the analysis was conducted using a Rtx-5 MS capillary column (60 m × 0.25 mm i.d., film thickness 0.25 μm). The temperature program was as follows: 40 °C for 5 min, ramped to 300 °C at 5 °C min^−1^, and held at 300 °C for 5 min. The chemical composition of the EOs was obtained by comparison with the NIST20 library and further confirmed by comparing retention indices with published data from other studies [17].

### 2.4. EOs Antibacterial Activity

In vitro assays were conducted using the agar diffusion method (Kirby-Bauer) and the determination of minimum inhibitory concentrations (MICs) as stated by Clinical and Laboratory Standards Institute (CLSI), with some modifications [18]. A total of 12 *S. pseudintermedius* isolates were obtained from dogs with pyoderma at various veterinary clinics in the Metropolitan Region, Chile, between 2018 and 2019. In addition, one *S. pseudintermedius* ATCC 49444 strain was included as a reference. To identify the strains, the isolates were cultured on Muller-Hinton Agar plates, incubated at 37 °C for 18 to 24 h, and identified using Gram staining. Selective isolation was performed on Mannitol Salt Agar, followed by confirmation of *S. pseudintermedius* through a biochemical identification panel. The bacterial isolates were stored at −80 °C in cryotubes. Bacterial suspensions were adjusted to 0.5 McFarland standard (≈1–2 × 10^8^ CFU/mL) before all antimicrobial assays [18].

For the agar diffusion assay, triplicate samples were prepared, with the following conditions: a control (disc with no antimicrobial agent), *C. alba* (disc with 5 µL CA-EO), *L. philippiana* (disc with 5 µL LP-EO), and Chlorhexidine (CHX) 2% (disc with 5 µL of antiseptic). The plates were incubated at 37 °C for 24 h, and the inhibition zones were measured using a caliper (Ubermann RM813 model). CHX 2% (DifemPharma, La Reina, Santiago, Chile) was used as the reference drug for both the agar diffusion assay and the MIC determination [19].

For MIC determination, 96-well microplates were prepared in a laminar flow hood. Wells 2 to 11 were loaded with 100 μL of Mueller–Hinton Broth (MHB, BD Difco™, Franklin Lakes, NJ, USA) supplemented with 10% dimethyl sulfoxide (DMSO), which was used to facilitate solubilization of the EOs and CHX during sample preparation and serial dilution. Well 12 served as the negative control and contained 200 μL of MHB with 5% DMSO. The procedure was performed in triplicate, with one plate prepared for each *S. pseudintermedius* isolate. For each plate, the first three rows were assigned to the EO treatment, and the last three rows were assigned to CHX control. In the first column, 1.78 μL of the corresponding antimicrobial agent was dissolved in 198.2 μL of MHB supplemented with 10% DMSO. Serial two-fold dilutions were then performed from column 1 to column 10, while column 11 was used as the positive growth control. Wells 1 to 11 were subsequently inoculated with 100 μL of bacterial culture, resulting in a final DMSO concentration of 5% and final antimicrobial concentrations ranging from 4.096 to 0.008 mg/mL in the test wells. A solvent control containing MHB with 5% DMSO and bacterial inoculum was included to verify that DMSO at this final concentration did not inhibit bacterial growth under the assay conditions. Plates were incubated for 18–20 h at 36.5 °C, and bacterial growth was analyzed using Magellan software V7.5 with a Tecan Infinite F50 microplate reader (Tecan, Männedorf, Switzerland) [18].

### 2.5. Hydrogel Physical and Rheological Characterization

Based on the formulation reported by Tomczykowa et al. with some modifications [20], three hydrogel formulations were prepared using an identical base composition: a hydrogel incorporating CA-EO (HCA), a hydrogel incorporating LP-EO (HLP), and a vehicle control without EO or antimicrobial agents (HVE). The hydrogels were prepared from a Carbopol-based matrix at room temperature (20 °C). Agitation was initially performed using a magnetic stir bar on a magnetic stirrer and manually, with a glass rod, once the base reached the desired consistency. For HCA and HLP, the EO was incorporated at the manual agitation step to reach a final concentration of 5%, and stirring was maintained until a homogeneous mixture was obtained. A total of 450 mL was prepared for each formulation, from which 150 mL were allocated for viscosity measurements, while the remainder was packaged into containers and stored under refrigeration (4 °C) for subsequent use.

Density was determined by weighing the content of a 1 mL aliquot from each hydrogel in an Eppendorf tube using an analytical balance (Radwag AS220/C/2) [21]. The pH was measured in triplicate using an AQUASEARCHER pH meter (AB23PH) (OHAUS, Parsippany, NJ, USA) equipped with an ST320 electrode, under controlled temperature conditions between 20 and 25 °C. For each measurement, a 0.5 mL aliquot of each hydrogel was diluted 1:10 with sterile bidistilled water. Viscosity was determined using a Brookfield viscometer (Model DV2T) equipped with spindle no. 3, set HA/HB. Measurements were performed for 1 min at rotational speeds ranging from 50 to 200 RPM.

### 2.6. Microbiological Quality Control of Hydrogel Formulations

To evaluate the efficacy and safety of the formulations during storage, samples previously kept under refrigeration (4 °C) for 24 h, 14 days, and 28 days were subjected to microbiological growth testing. The hydrogel was incubated for 24 h at 36.5 °C on Mueller–Hinton agar and Columbia blood agar plates, with inoculation performed using both streak plate (depletion) and lawn (spread) methods.

### 2.7. Hydrogel Stability

Stability testing was performed through accelerated physical, accelerated thermal, and long-term evaluations. Physical stability was assessed through an accelerated centrifugation test, in which 1 mL aliquots of each formulation were transferred into 1.5 mL Eppendorf tubes and centrifuged at 13,000 RPM for 10 min at room temperature (20 °C). Additionally, accelerated thermal stability was evaluated by placing the samples in an oven at 50 °C for 120 h. Evaluations were conducted every 24 h, recording the number of phases, color, and aroma. The pH was measured at the beginning and at the end of the assay. The pH was measured to ensure a physiological canine pH, reported to range from 4.40 to 8.18 in dogs older than 12 weeks [22]. Table 1 presents the predefined criteria used to assess aroma.

For the long-term stability test, 1 mL aliquots were prepared in 1.5 mL Eppendorf tubes and stored under refrigerated conditions (4 °C) for 6 months. After this period, appearance, physical behavior, and aroma were evaluated. The study remains in progress, with a total duration of twelve months [23].

**Table 1:** Aroma intensity scale.

Score	Description
0	Odorless.
1	Almost imperceptible aroma.
2	Mild aroma, perceptible at a short range (<10 cm) from the microcentrifuge tube.
3	Moderate aroma, perceptible at a moderate range (10–20 cm) from the microcentrifuge tube.
4	Strong aroma, easily perceptible at distances >20 cm from the microcentrifuge tube.

Relative scale developed adapting the scale proposed by Chen [24].

### 2.8. Pre-Clinical Safety Test

Before the efficacy test in dogs, a preliminary safety test was performed in mice. For this purpose, eighteen healthy male and female *Mus musculus* BALB/c mice were obtained from the Experimental Platform of the Faculty of Odontology, Universidad de Chile. BALB/c mice of both sexes were included, according to availability for each prototype evaluated, to ensure biological variability (n = 10 males and n = 8 females; total n = 18). The animals were allocated into three groups (n = 6 per group). According to the formulation administered, the groups were as follows: (i) HCA, (ii) HLP, and (iii) HVE. All animals were handled in accordance with the guidelines of the Ethics Committee of the Faculty of Dentistry, Universidad de Chile (CICUA approval in 4 June 2025 code: 24814–ODO–UCH–e3).

All mice were housed in a conventional facility in a temperature-controlled room with a 12 h light/12 h dark cycle at the Faculty of Dentistry, Universidad de Chile. All animals were weighed, and the hair in a 1 × 2 cm area on the dorsal side was removed to allow direct application of the formulations to the skin. Each application consisted of 50 µL of hydrogel, delivering 2.3 mg of EO per application. Weight loss, food and water intake, appearance, spontaneous behavior, response to handling, and body temperature were monitored after the first application. A second dose of each formulation was administered after an 8 h period, followed by the same monitoring. For days 2, 3, and 4 of the irritation test, the same application protocol was performed. On day 5 of the irritation test, all animals were euthanized by controlled CO_2_ exposure. Immediately afterwards, skin samples from the treated dorsal area were collected and transferred to 15 mL centrifuge tubes containing 10% formaldehyde [25,26].

#### 2.8.1. Skin Irritation Measurements

A skin irritation test was conducted to assess the degree of skin irritation associated with hydrogel application, evaluated at 1, 4, and 8 h post-application. Two criteria were used to describe skin irritability, as detailed in Table 2.

#### 2.8.2. Ex Vivo Histological Analysis

Tissue sections of 5 μm were deparaffinized in xylene (Biopack, Pershore, WR10 2TA, England) for 15 min, rehydrated through a graded series of ethanol (100%, 95%, 80%, 70%, and 50%) for 5 min each, and stained with hematoxylin and eosin (H&E, Biopack, Pershore, WR10 2TA, UK) for 5 min. Subsequently, excess dye was rinsed under running water for 2 min, and sections were dehydrated using increasing concentrations of ethanol (50%, 70%, 80%, 95%, and 100%). The slides were then mounted using a microscopy mounting medium (Biopack, Pershore, WR10 2TA, UK). Once dried, five representative fields per group were visualized and analyzed under bright-field microscopy using an Olympus microscope (Olympus, Tokyo, Japan). Images were captured with a DFC 295 digital camera (Olympus, Tokyo, Japan) 4× objective (total magnification 40×). Two specialized pathologists blindly analyzed histological sections to evaluate epithelial changes, including cellularity, nuclear-to-cytoplasmic ratio, inflammatory infiltrate, and blistering, among others, according to the pathognomonic histological features described for this animal model [27].

### 2.9. Microbiological Evaluation in Dogs

#### 2.9.1. Study Design and Ethical Approval

A preliminary microbiological evaluation was performed in dogs diagnosed with superficial bacterial pyoderma and treated with topical formulations. A total of 32 client-owned dogs diagnosed with superficial bacterial pyoderma were included in the study. Animals were recruited from different clinical settings in Santiago, Chile, including private veterinary clinics and university-associated veterinary services (CICUA approval code: ID14/2024, 28 June 2024).

Dogs were allocated into four treatment groups of eight animals each: HCA, hydrogel loaded with HLP, HVE, and a commercial antiseptic control (Inveclor^®^, Drag Pharma, Santiago, Chile). Available age data indicated a range between 1 and 10 years, with both sexes represented. Lesions associated with superficial pyoderma were mainly located on the trunk, abdomen, and intertriginous areas, and were characterized by epidermal collarettes, crusts, pustules, and intertrigo.

#### 2.9.2. Microbiological Sampling and Bacterial Identification

Microbiological samples were collected from active skin lesions using sterile cotton swabs on Day 1 (baseline) and Day 21 (end of treatment), following principles commonly applied in the microbiological diagnosis of canine superficial pyoderma and veterinary dermatology infections [28,29]. Before sampling, the lesion surface was gently cleared of superficial debris using sterile gauze without applying antiseptic solutions to avoid interference with bacterial recovery. The sterile swab was rolled across the lesion surface to collect exudate and bacterial material. Samples were immediately transported under refrigerated conditions to the MICROVET Diagnostic Laboratory (Faculty of Veterinary and Animal Sciences, University of Chile) for microbiological processing. Samples were inoculated onto blood agar plates and incubated at 37 °C for 24–48 h. Colonies compatible with *staphylococci* were subcultured to obtain pure isolates. Bacterial identification was subsequently performed using the VITEK^®^ automated microbial identification system (bioMérieux, Durham, NC, USA), allowing species-level identification of *S. pseudintermedius* [30]. The presence or absence of *S. pseudintermedius* was recorded for each sample. At baseline (day 1), all dogs included in the study showed positive identification of *Staphylococcus* spp., confirming the bacterial etiology of the lesions. Bacterial identification was classified as: *S. pseudintermedius*; *Staphylococcus aureus*; other *Staphylococci* (*S. capitis*, *S. warneri*); no bacteria detected. For the purposes of this analysis, microbiological results were simplified into a binary outcome: presence: identification of any *Staphylococcus* species; or absence: no cocci identified in the culture. Additionally, bacterial burden was assessed by quantifying colony-forming units (CFU) obtained from lesion swab cultures on day 1 (baseline) and day 21 (end of treatment). At baseline, all dogs presented bacterial counts exceeding 300 CFU per swab, corresponding to a “very high” (uncountable) bacterial load, thereby confirming a homogeneous starting point across all treatment groups. On day 21, bacterial load was classified into three categories according to CFU counts: non-significant: 0–15 CFU/swab; countable: 15–300 CFU/swab; uncountable: >300 CFU/swab [31,32].

### 2.10. Statistical Analysis

Significant differences in agar diffusion, as well as hydrogel density and pH, were evaluated using a two-way ANOVA (ordinary) in GraphPad Prism (Version: 10). Row effects, column effects, and their interaction were included.

For the preliminary microbiological evaluation in dogs, categorical outcomes were analyzed descriptively and inferentially. Bacterial detection at Day 21 was analyzed as a binary outcome, defined as presence or absence of detectable cocci/*Staphylococcus* spp. in culture. Bacterial load was analyzed as an ordinal categorical outcome according to predefined CFU-based categories: non-significant bacterial load (0–15 CFU/swab), countable bacterial load (16–300 CFU/swab), and uncountable bacterial load (>300 CFU/swab). Due to the exploratory nature of the study, the small sample size, and the categorical structure of the outcomes, differences among treatment groups were evaluated using the Fisher–Freeman–Halton exact test, an extension of Fisher’s exact test for contingency tables larger than 2 × 2. In addition, bacterial load was also dichotomized for clinical interpretation into reduced bacterial load, defined as the non-significant category, versus persistent bacterial load, defined as countable or uncountable growth. Statistical significance was defined as *p* < 0.05 for all analyses.

## 3. Results

### 3.1. Chemical Characterization

A total of 62 molecules were determined by GC-MS in both EOs (Table 3), 16 of which were not identified, representing 4.2% and 0.95% of the total chromatogram area for CA-EO and LP-EO, respectively. 43 compounds were successfully identified in the CA-EO, of which sabinene and eucalyptol were the major compounds with 13.95 and 12.05%. In contrast, only 20 compounds were successfully identified in LP-EO, and the major compounds were linalool (37%), eucalyptol (24.54%), and safrole (11.42%). 17 compounds were found in both EOs, hence EOs differ in 73% of the volatile compounds summarized in Table 3.

### 3.2. EOs Antibacterial Activity

A total of 12 *S. pseudintermedius* isolates obtained from dogs were tested alongside *S. pseudintermedius* ATCC 49444 used as a reference. CHX 2% was used as a reference antiseptic. The most susceptible strain to CA-EO was SP 9, and to LP-EO was SP 13, showing inhibition zones of 10.38 and 10.80 mm, respectively (Table 4). The least susceptible strain for both EOs was SP 8. In most cases, inhibition by CA-EO was greater than that of LP-EO at the concentrations tested. Both EOs exhibited nearly half the activity of the reference antiseptic against the reference strain SP ATCC 49444. The inhibition zone for most isolates was greater than 6 mm, except for SP 8 treated with LP-EO, which showed an inhibition diameter of 4.92 mm. The antibacterial activity of CA-EO was higher than that of LP-EO, as the mean inhibition zone diameters per EO were 9.09 mm for CA-EO and 6.76 mm for LP-EO, representing 37% and 28% of the activity registered for the reference drug CHX. Statistical analysis revealed significant effects (*p* < 0.05) of antimicrobial strain, and their interaction. CHX showed significantly greater antibacterial inhibition than both EOs across all strains, while differences between CA-EO and LP-EO were strain-dependent, being significant only for SP8, SP9, SP12 (higher for CA-EO) and SP13 (higher for LP-EO).

Regarding the MIC (Table 5), the results showed much higher MIC values for the EOs than the reference antimicrobial (CHX). Almost every EO MIC was 4.096 mg mL^−1^ or higher (not determined), except for LP-EO against SP 12, whose MIC was 2.048 mg mL^−1^. All SP strains were susceptible to CHX with MICs ranging from 0.064 mg mL^−1^ to 0.128 mg mL^−1^.

### 3.3. Hydrogel Physical and Rheological Characterization

In this study, three hydrogels were formulated: HCA (containing CA-EO), HLP (containing LP-EO), and HVE (vehicle). Both EO-loaded formulations were semisolid (score 4, Table 1), easily extensible, white, homogeneous, and with no evidence of phase separation. HVE had similar characteristics but without visible coloration.

Density values of the hydrogel formulations exhibited minimal variation, with a range from 1.02 to 1.05 g·mL^−1^ (Supplementary Material S1), indicating a consistent bulk composition among samples. The initial mean pH (24 h after formulation) was 7.68, 7.74, and 7.72 for HCA, HLP, and HVE, respectively.

Upon viscosity measure, the lowest viscosity values were obtained at 200 RPM in all cases, measuring 1.248 Pa·s, 1.680 Pa·s, and 1.760 Pa·s, with respective torque values of 31.2%, 42.0%, and 44.0% for HCA, HLP, and HVE (Supplementary Material S2). All torque values obtained within the 50–200 RPM range exceeded the 10% threshold, with the lowest value corresponding to HCA at 18.2% at 50 RPM. Therefore, all measurements fall within the valid operating range of the instrument [33].

### 3.4. Microbiological Quality Control

To establish the microbiological safety of formulations, cultures were examined individually under a laminar flow hood with the plates opened to detect microbial growth (colony formation). None of the plates inoculated with the hydrogels (HCA, HLP, HVE) showed evidence of microbial growth 1, 14, and 28 days after inoculation of the formulations, regardless of the inoculation method used (Supplementary Material S3).

### 3.5. Hydrogel Stability

Thermal stability was evaluated through the observation of the formation of two or more phases after thermal treatment. Evidence of instability was most pronounced in HCA, which exhibited progressive phase separation: three distinct layers after 24 h, with a more pronounced separation than that observed in HLP (Supplementary Material S4). Although HLP developed two phases at 24 h, the effect was comparatively less evident (Supplementary Material S5). This type of physical change is relevant from a formulation perspective, as alterations in consistency or phase organization may affect the uniformity in the distribution of the EO within the hydrogel matrix. Consequently, the amount of active compounds delivered per application could vary from the intended dose, affecting the consistency of topical administration and the comparability of results between applications. Nevertheless, when phase separation was observed, the separated phases could be re-homogenized by manual agitation. This was considered in the standardized user manual used during the canine study, which included shaking the formulation before each application. Under these controlled conditions, the samples maintained a spreadable and applicable consistency after shaking, allowing their administration during the in vivo study.

At the end of the assay, all EO-loaded formulations showed at least one separate phase at the bottom of the microcentrifuge tube, which remained static in every case. In HCA and HLP, this lower phase was visually distinguishable due to its transparent appearance, contrasting with the white hydrogel matrix. In HVE, phase separation was not detected. HCA lost consistency over time, in comparison with the other two formulations (Supplementary Material S4).

Regarding the odor of the formulations (Supplementary Material S5), aroma was included as it provides indirect, yet relevant, information concerning EO-loaded formulations. Aroma intensity was assessed using a study-specific arbitrary scale, while the characteristic EO aroma was evaluated qualitatively, allowing the detection of its maintenance, loss, or modification into a non-characteristic odor. Under thermal stress, neither HCA nor HLP showed abnormal odor changes, and the characteristic aroma of each EO was maintained throughout the assay. However, aroma intensity decreased from score 4 to score 3, indicating a change from strong to moderate aroma; therefore, a partial loss of volatile aromatic compounds under accelerated conditions cannot be ruled out. HVE remained odorless throughout the evaluation.

This is relevant, as several volatile constituents are also linked to antibacterial activity. In this regard, the hydrogels were formulated with EO at 5% *v*/*v*, whereas the microdilution assays showed antibacterial activity mainly at approximately 0.445% *v*/*v*, equivalent to 4.096 mg/mL. Therefore, potential losses of volatile active compounds would need to be substantial to reduce the EO concentration below the in vitro activity threshold observed in this study.

The initial pH values (Supplementary Material S6) were 7.75, 7.81, and 7.79 for HCA, HLP, and HVE, respectively. After the evaluation period, these values decreased to 7.35, 7.66, and 7.72. The largest decrease was recorded for HCA (ΔpH = 0.40), whereas HVE exhibited the smallest variation (ΔpH = 0.07). These results highlight the importance of maintaining pharmaceutical formulations under suitable storage conditions, including controlled temperature, protection from sunlight, and appropriate humidity [34].

None of the formulations exhibited phase separation during the centrifugation test, nor any other visually appreciable changes (Supplementary Material S7).

Regarding long-term stability, under refrigerated storage at 4 °C for six months, HCA exhibited marked physical instability, characterized by phase separation into three distinct layers and a substantial loss of structural integrity, ultimately adopting an aqueous-like consistency (Supplementary Material S8). Despite this, its aromatic characteristics remained unchanged, with maximum intensity (score 4). HLP showed a subtle phase separation characterized by a thin, transparent peripheral layer inside the centrifuge tube. Its consistency did not show significant changes, while both its characteristic aroma and intensity were preserved. H-VE did not exhibit detectable phase separation, retained its initial consistency, and remained odorless. These long-term findings indicate that, under the usual storage conditions defined for the hydrogels, both EO-loaded formulations preserved their characteristic aroma and maximum aroma intensity score. However, additional studies are required to determine whether this sensory observation correlates with retained chemical stability or biological activity. Future studies should directly quantify the chemical stability of the main EO marker compounds within the hydrogel matrix and evaluate the antibacterial activity of freshly prepared and stored EO-loaded hydrogels.

### 3.6. Pre-Clinical Safety Test

**Figure 1:**
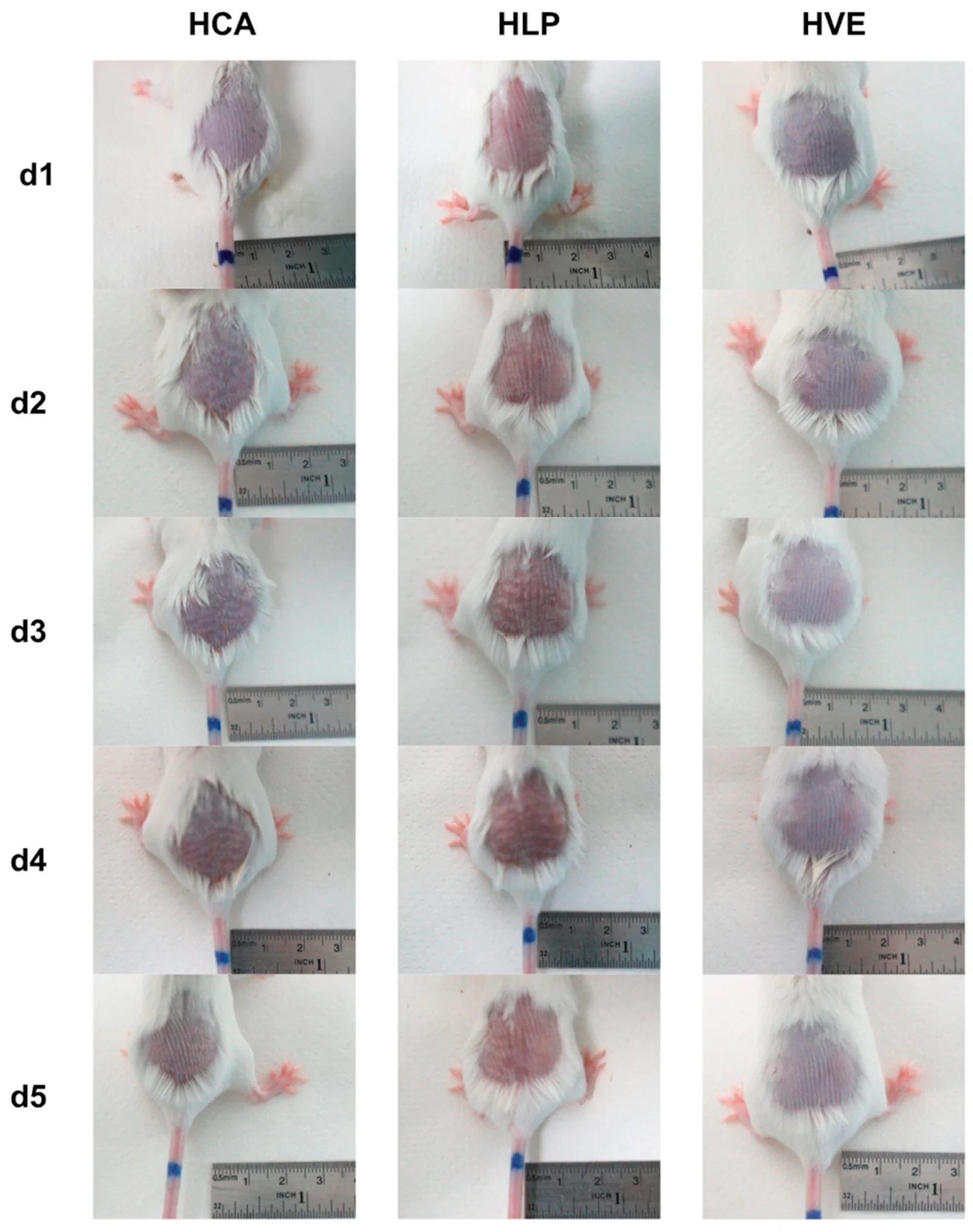
Representative photographs of the dorsal skin of mice from the experimental groups during the 5-day dermal safety assessment after repeated topical application of the formulations. Images were taken daily (**d1**–**d5**) for mice treated with HCA (hydrogel containing CA-EO), HLP (hydrogel containing *L. philippiana* EO), and HVE (vehicle hydrogel without antimicrobial agent). A more evident reddish coloration of the treated area was visually observed in the HLP group throughout the evaluation period, whereas HCA and HVE showed a milder and more homogeneous appearance. Although slight differences in skin coloration can be observed among photographs, these were attributed to lighting conditions, residual hair, and exposure of the shaved dorsal skin area, and were not considered compatible with treatment-related erythema. All animals scored 0 for erythema and edema throughout the evaluation period.

The clinical status of each mouse was monitored daily throughout the treatment period. No alterations were observed in general appearance (Figure 1), food and water intake, clinical signs, body temperature, or behavior, resulting in a score of 0 across all evaluated parameters. Statistical analysis showed no significant effects associated with the formulation (*p* = 0.9445), time (*p* = 0.2319), or their interaction (*p* = 0.9997).

Body weight variations remained within the expected physiological range (<5%) and were not considered clinically relevant. Only one animal exhibited a weight loss exceeding 5%, without associated clinical or behavioral changes. In all other cases, minor fluctuations were attributed to variations in food and water intake or measurement variability, with no impact on animal welfare.

Both the in situ evaluation and the retrospective photographic assessment revealed no alterations in skin coloration compatible with erythema or increases in the size of the application site indicative of inflammation, for any of the formulations tested, resulting in a score of 0 in the erythema and edema evaluation protocol.

**Figure 2:**
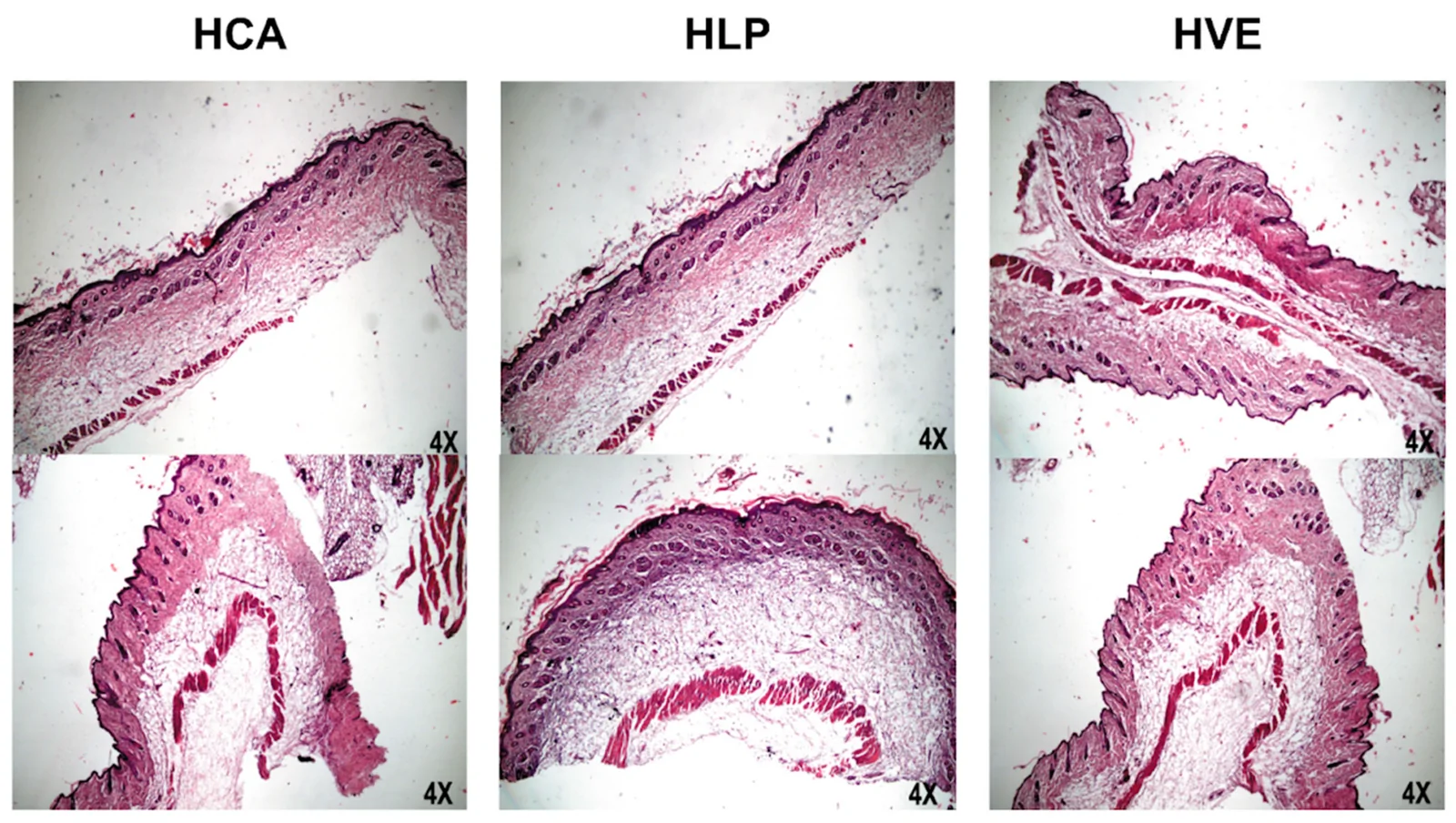
Representative images of homogenized tissue samples obtained from mouse tissues, grouped according to hydrogel treatment, and examined by light microscopy using a 4× objective (total magnification 40×). HCA: hydrogel containing *C. alba* essential oil; HLP: hydrogel containing *L. philippiana* essential oil; HVE: vehicle hydrogel without antimicrobial agent.

All animals maintained scores under 1 on the Morton & Griffiths scale [35] throughout the experiment. Neither analgesic administration nor early euthanasia was required. Ex vivo skin samples were collected from all individuals for histological analysis. The skin exhibited a generally preserved architecture across all treatments, with a clear differentiation between epidermis and dermis (Figure 2). No signs of necrosis, inflammatory infiltrates, or structural alterations were observed at the evaluated total magnification (40×).

### 3.7. Microbiological Evaluation in Dogs

To provide preliminary evidence of antimicrobial activity in the target species, a microbiological evaluation was conducted in client-owned dogs diagnosed with superficial pyoderma. Dogs were initially allocated into four treatment groups. However, two dogs from the vehicle hydrogel group discontinued the study before the final microbiological evaluation; therefore, the final microbiological analysis included eight dogs in the HCA group, eight dogs in the HLP group, six dogs in the HVE group, and eight dogs in the Inveclor^®^ group. At baseline, all dogs were culture-positive for *Staphylococcus* spp., with *S. pseudintermedius* being the predominant species identified. On Day 21, *S. pseudintermedius* remained the most frequently detected species; however, changes in bacterial identification were observed in some animals, including transitions between *S. pseudintermedius* and *S. aureus*, shifts toward other staphylococcal species, and cases in which no cocci were detected in culture (Table 6).

At Day 21, bacterial elimination, defined as absence of detectable cocci in culture, was observed in the active treatment groups but not in the vehicle hydrogel group. The highest elimination rate was observed in the Inveclor^®^ group, with absence of detectable cocci in 4 of 8 dogs (50.0%). Both EO-loaded hydrogel groups showed bacterial elimination in 2 of 8 dogs (25.0%) each. In contrast, all dogs in the vehicle hydrogel group remained culture-positive at Day 21 (Table 7). Comparison of bacterial presence/absence among treatment groups using the Fisher–Freeman–Halton exact test did not reveal statistically significant differences (*p* = 0.2576).

Additionally, bacterial load was categorized on Day 21 to provide a more detailed assessment of microbiological response (Table 8). The highest proportion of dogs reaching the non-significant bacterial load category was observed in the HLP group (6/8; 75%), followed by the Inveclor^®^ group (4/8; 50.0%) and the HCA group (3/8; 37.5%). In contrast, no dogs in the vehicle hydrogel group reached this category.

Intermediate bacterial loads were most frequently observed in the vehicle group (4/6; 66.7%). The lowest proportion of dogs remaining in the uncountable category was observed in the HLP group (1/8; 12.5%), whereas higher proportions were observed in the HCA group (3/8; 37.5%), Inveclor^®^ group (3/8; 37.5%), and vehicle group (2/6; 33.3%) (Table 8). Comparison of bacterial load distribution among treatment groups using the Fisher–Freeman–Halton exact test did not reveal statistically significant differences (*p* = 0.1139).

**Figure 3:**
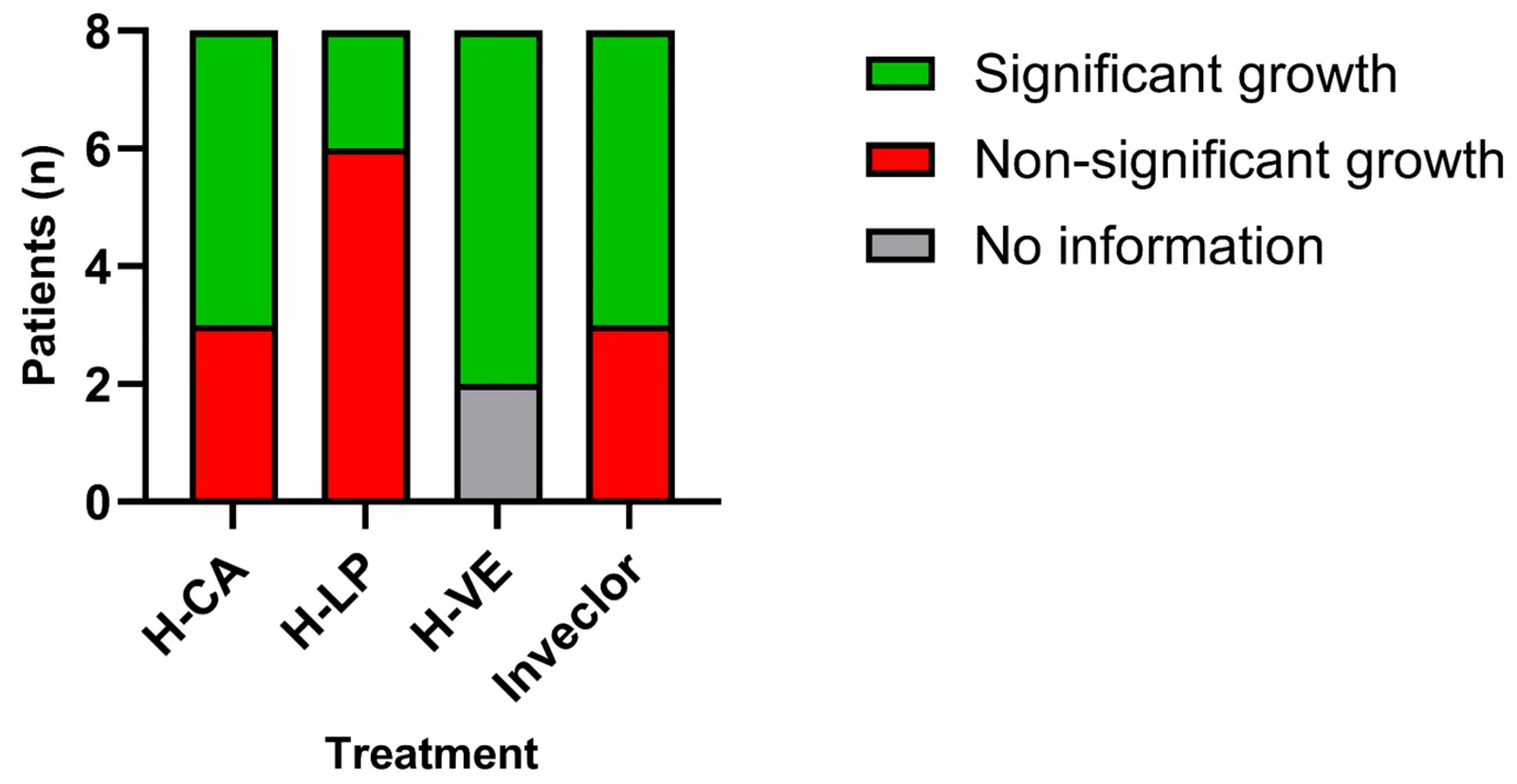
Significant growth (>15 CFU) or non-significant growth of cocci in final microbiological cultures (Day 21) across treatment groups (*p* = 0.0232). Gray indicates unavailable data. HCA: hydrogel containing *C. alba* essential oil; HLP: hydrogel containing *L. philippiana* essential oil; HVE: vehicle hydrogel without antimicrobial agent.

At Day 21, bacterial elimination, defined as absence of detectable cocci in culture, was observed in the active treatment groups but not in the vehicle hydrogel group. The highest elimination rate was observed in the Inveclor^®^ group, with absence of detectable cocci in 4 of 8 dogs (50.0%). Both EO-loaded hydrogel groups showed bacterial elimination in 2 of 8 dogs (25.0%) each. In contrast, all dogs in the vehicle hydrogel group remained culture-positive at Day 21 (Figure 3; Table 7). However, comparison of bacterial elimination rates among treatment groups using Fisher–Freeman–Halton exact test did not reveal statistically significant differences (*p* = 0.116) (Figure 3).

Fisher’s exact test revealed significant differences among treatment groups (*p* = 0.0232), with H-LP showing the lowest proportion of significant bacterial growth at Day 21 (2/8; 25%) (Figure 3).

## 4. Discussion

### 4.1. EOs’ Chemical Characterization

The present study demonstrates the feasibility of developing hydrogel-based delivery systems incorporating EOs from the Chilean endemic species *C. alba* and *L. philippiana*, with potential application in the topical management of canine pyoderma. This work integrates phytochemical characterization, antimicrobial evaluation, pharmaceutical formulation, and preclinical safety assessment, providing a comprehensive approach toward the development of plant-based veterinary therapeutics.

A total of 62 compounds were identified by GC-MS (Table 3) for the samples analyzed in this work. As previously described, the major compounds in CA-EO, sabinene and eucalyptol, corresponded to 13.95% and 12.05% of the total chromatogram area, respectively, while linalool, eucalyptol, and safrole were the main compounds in LP-EO, corresponding to 37.91%, 24.54%, and 11.42% of the total chromatogram area. In a previous study by Touma et al., the authors reported the chemical composition of CA-EO obtained from *C. alba* leaves from Chicauma, Lampa [16]. Although the sampling location is close, the major compounds of CA-EO were α-terpineol, eucalyptol, and β-phellandrene (24.96%, 21.63%, and 14.84%, respectively), which differs from the data summarized in Table 1. This difference in the chemical composition of both CA-EOs could be attributed to meteorological factors such as water accessibility and solar light [36]. Sabinene and eucalyptol are commonly found in EOs from *C. alba* with concentrations ranging from 6.8 to 13.5% for sabinene and from 1.9 to 23.3% for eucalyptol in samples from the central region of Chile [37,38,39,40]. The chemical composition of *L. phillipiana* leaves EO from the Valdivian forest was reported by Bruna et al. in 2022 [15]. The authors found that the major compounds were eucalyptol (27.7%), linalool (27.6%), and isosafrole (19.5%), which is similar to the data summarized in Table 3.

The chemical characterization of the EOs confirmed distinct phytochemical profiles between *C. alba* and *L. philippiana*. The main compounds identified in CA-EO were sabinene and eucalyptol, whereas LP-EO was mainly composed of linalool, eucalyptol, and safrole. Several terpenoid compounds commonly present in EOs have been associated with antimicrobial activity, particularly through effects on bacterial membranes, cytoplasmic integrity, ion permeability, and cellular homeostasis [41,42]. However, EOs are complex mixtures, and their biological activity cannot be attributed exclusively to individual major compounds. Instead, synergistic or antagonistic interactions among minor and major constituents may influence their antimicrobial performance [41,42]. Therefore, although the composition of the oils provides a plausible basis for antibacterial activity, the contribution of specific compounds should be interpreted cautiously.

### 4.2. EOs Antibacterial Activity

It is important to note that the antimicrobial activity of any natural product is a consequence of its chemical composition and, more importantly, the synergy between these compounds. Eucalyptol, linalool, sabinene, and safrole have been studied for their antibacterial activity against *S. aureus*, *Staphylococcus epidermidis*, *P. aeruginosa*, *Clostridium perfringens, Streptococcus pyogenes*, and *Haemophilus influenzae* [12,43,44,45,46]. Eucalyptol can compromise the integrity and function of the bacterial membrane and inhibits film formation and quorum sensing [1,14]. Membrane permeabilization allows other antibacterial compounds to enter the bacteria, thereby enhancing the efficacy of antibiotics [43]. On the other hand, linalool interrupts the energy production of *P. aeruginosa* by inhibition of hexokinase, cytokines, and pyruvate quinase activity, key enzymes of the glycolytic pathway, hence lowering the ATP concentration; it also interferes with the pentose-phosphate cycle, altering the cell’s redox state [46].

Pyoderma in dogs is commonly caused by SP infections [2]. Recently, with the rise of multidrug-resistant strains, research on MRS has emerged as a relevant topic in health management [2,4]. Antimicrobial prescribing to treat canine pyoderma is a standard practice; however, accurate identification of the bacterial cause is essential to prevent inappropriate antimicrobial use [29]. When comparing the antibacterial effect of the EOs tested (Table 4 and Table 5), CA-EO had a larger inhibition halo against SP 9, while LP-EO had the largest effect against strain SP 13 (Table 4). This difference in inhibition halo is to be expected due to the different chemical composition of the EOs (Table 3). These differences may be associated with variations in EO composition; however, the contribution of individual compounds versus synergistic effects requires further investigation, and SP 13 towards linalool, the major compounds of CA-EO and LP-EO, respectively; nevertheless, it is also possible to theorize that the greater sensitivity was likely due to the synergy between all compounds present in the EOs. To further elucidate the contribution of the major compounds to the antibacterial activity, more research is needed. Both EOs had a significantly lower inhibition halo (*p* < 0.05) than the reference antiseptic CHX.

Results concerning CA-EO antibacterial activity (Table 5 and Table 6) are in accordance with the data reported by Avello et al. [37]. The authors tested the antibacterial activity of CA-EO against *S. aureus*, reporting inhibition halos between 6 and 9 mm, but the specific strain evaluated was not specified. Touma et al. reported inhibition halos larger than 20 mm when using CA-EO against clinical isolates and the ATCC 25923 strain of *S. aureus,* which is higher than the inhibition halos reported in the present work against *S. pseudintermedius* (Table 4) [16]. A similar phenomenon is observed regarding MIC values (Table 5); a much lower MIC was reported against *S. aureus* (0.0019 mg mL^−1^). These discrepancies may be explained by the variation in bacterial species evaluated, as well as by differences in the chemical composition of the EOs employed by Touma et al. [16]. As for LP-EO’s antibacterial activity (Table 5 and Table 6), inhibition halos are comparable to those reported by Toledo et al. for *S. aureus* and *S. epidermidis* with values ranging from 7 to 8 mm. Similarly, MIC values of LP-EO against *S. aureus* are also much lower (0.0032 mg mL^−1^) than those presented in Table 5 against SP strains [47].

CA-EO MIC results (Table 5) show a slightly lower EO inhibitory concentration for the SP 8 and SP 9 (4.096 mg L^−1^), which were the only strains to present better MIC than the SP ATCC 49444 (reference strain). Similarly, CA-EO showed that only for SP 12, the MIC was half the MIC value of the reference strain. These findings are among the values reported for other EOs, such as black pepper (0.21 mg mL^−1^) and *Copaifera officinalis* (26.52 mg mL^−1^) against *S. aureus* [48]. Due to the large chemical variability of natural products such as EOs, and the genetic variability of the bacteria tested, the inhibition halos and MIC values vary widely between scientific investigations [49]. Although the antibacterial activity of the EOs was lower than that of CHX, their incorporation into a hydrogel system may enhance local bioavailability and retention at the application site, potentially compensating for their lower intrinsic potency.

Ebani et al. tested 9 EOs against multi-drug-resistant *S. aureus, P. aeruginosa,* and *S. pseudintermedius*. Of the EOs tested, *Origanum vulgare* and *Salvia sclarea* showed better performance against multi-drug-resistant SP [50]. Eucalyptol was one of the major chemical components of *O. vulgaris* EO, corresponding to 22% of the EO, similar to LP-EO. The authors reported that *Ocimum basilicum* had a 46% of linalool and showed similar inhibition halos to CA-EO (Table 4) [50].

Previous reports place the activity of several EOs against *S. pseudintermedius* within a comparable MIC range [3]. In contrast, the higher MICs reported for isolated linalool suggest that whole EOs may act through synergistic interactions among their components [51,52].

The in vitro antibacterial activity observed against *S. pseudintermedius* was moderate. Both EOs produced measurable inhibition zones, but their activity was lower than that of CHX, and MIC values were generally high. This is consistent with the broader evidence showing that CHX has strong support as a topical antimicrobial for canine bacterial skin infections, whereas alternative topical agents often show more variable levels of evidence and efficacy [53]. Thus, these results should not be interpreted as evidence of superiority over CHX. Rather, they indicate that both EOs possess intrinsic antibacterial activity that may justify further evaluation when incorporated into a topical delivery system.

This distinction is relevant because the clinical value of EO-based formulations may not depend solely on the potency of the free oil measured in conventional in vitro assays. Topical delivery systems may influence local bioavailability, residence time, compound diffusion, and interaction with the skin microenvironment. Hydrogels are widely used as wound and topical matrices because they can maintain hydration, improve contact with the application site, and serve as carriers for active compounds. However, in the present study, release kinetics, skin permeation, local bioavailability, and retention of EO components were not directly evaluated; therefore, no conclusion can be drawn regarding enhanced delivery of the active compounds by the hydrogel matrix.

### 4.3. Hydrogel Physical and Rheological Characterization

Carbopol^®^ hydrogels typically have densities around 1 g mL^−1^ due to their aqueous nature [54], which is consistent with the density values for all formulations tested in this work. The absence of significant differences between density values shows that the EO incorporation was homogeneous and did not change the mass per unit volume. pH values (Supplementary Material S1) show no significant difference between each EO-loaded formulation and HVE, but the pH of HCA was statistically lower than the HLP pH value (*p* < 0.05). The pH of a dog’s skin may vary with its age, gender, and breed [55], and strongly depends on apocrine gland secretion [56]. The most reported pH range is 5.5–7.2, but studies have shown an elevated pH value ranging between 4.84 and 9.95 [56]. Given that the pH of all formulations is close to pH 7.7, the EO-carrying formulations are safe to use on dog skin under this parameter.

As shown in Supplementary Material S2, HCA exhibits lower viscosity and torque than HLP along the entire RPM interval, while HLP has a behavior comparable to HVE. The chemical interactions between the formulation components determine their internal friction and, hence, their viscosity and torque. Interestingly, HLP has a very similar behavior to HVE regarding these rheological parameters. This indicates that the addition of LP-EO does not sufficiently affect the internal interactions to alter the formulation’s viscosity. In contrast, the rheological behavior of HCA is markedly different. The major components of LP-EO (linalool, eucalyptol, and safrole) may interact with the Carbopol^®^ matrix via hydrogen bonds, resulting in a higher viscosity.

Based on the results, and considering the satisfactory and consistent performance of HLP, comparable to the vehicle across all tests, storage under refrigerated conditions at 4 °C is recommended, as intended by its design.

Although the EO-loaded hydrogels showed promising physicochemical behavior, particularly HLP, the antibacterial activity of the formulated hydrogels was not directly evaluated through standardized in vitro assays. This represents a limitation, as the hydrogel matrix may affect EO diffusion, release, and bioavailability; however, these parameters were not experimentally assessed in the present study. Therefore, future studies should include direct antibacterial testing of EO-loaded hydrogels This represents a limitation, as the hydrogel matrix may influence EO diffusion, release, and bioavailability. In addition, antibacterial activity after storage was not directly assessed using stored hydrogel samples, and the chemical stability of specific EO marker compounds within the hydrogel matrix remains to be determined. Therefore, future studies should include direct antibacterial testing of EO-loaded hydrogels before and after storage, together with chemical monitoring of the main monoterpene markers of each EO, to confirm long-term stability and bioactivity retention.

The physicochemical characterization showed that both EO-loaded hydrogels could be formulated successfully, although formulation performance differed between oils. HLP showed more favorable physical behavior, with rheological characteristics closer to the vehicle and better long-term stability than HCA. This difference is relevant from a pharmaceutical perspective, as topical formulations require adequate consistency, stability, and applicability to support reproducible administration and owner adherence. In this regard, formulation characteristics are not only technological attributes but also relevant determinants of practical clinical use in dermatological therapy [51,52]. The stability results therefore support HLP as the more promising formulation from a technological standpoint.

### 4.4. Microbiological Safety of the Formulations

The Chilean Servicio Agrícola y Ganadero (SAG) establishes guidelines for registering new pharmaceutical products for veterinary use [57]. To ensure that the hydrogel formulations comply with these safety requirements, microbiological safety was tested. Supplementary Material S1 (Table S1) shows that none of the formulations presented pathogen growth at 1, 14, or 28 days following inoculation of 10 µL of each formulation. These results show that the formulations proposed in this work are resistant to pathogen growth for up to 4 weeks, making them a promising pharmaceutical product.

Microbiological quality control showed no detectable microbial growth in the tested formulations during storage, supporting their microbiological safety under the evaluated conditions. This is an important requirement for topical veterinary products, particularly those intended for use on compromised skin. In addition, the pH values of the formulations were compatible with the reported physiological characteristics of canine skin, which differs from human skin and may vary among species, anatomical sites, and individuals [56]. This supports the suitability of the formulations for topical application in dogs.

### 4.5. Hydrogel’s Stability

As hydrogels are prone to water loss over time, it is crucial to know if the formulation is susceptible to thermal instability due to water loss. Results summarized in Supplementary Materials S4–S6 show changes in the formulations due to thermal stress. HCA consistency showed a decrease after 24 h, which is to be expected because of water loss at 50 °C. Regarding the aroma of each formulation, HCA maintained an intensity score of 4 until the 72 h mark; in contrast, HLP presented a decreased intensity score after 24 h of thermal treatment. Loss of aroma is relevant in terms of future clinical applications since it is directly related to the concentration of the bioactive compounds in the formulation. These results show the importance of maintaining pharmaceutical formulations under suitable storage conditions (temperature, sunlight, humidity, etc.) [34]. In terms of pH, statistically significant differences were observed only for HCA, where the pH value decreased by 0.4 units during the temperature assay (Supplementary Material S6).

The long-term stability test showed evident differences between the HCA and HLP formulations, with the HCA formulation being more unstable than the HLP formulation. HCA lost its consistency and three phases were formed under refrigerated conditions after 6 months of storage (Supplementary Material S8). Despite this, HCA retained a strong and characteristic *C. alba* aroma (Supplementary Material S8), maintaining its aroma score. However, further studies are required to determine whether this observation correlates with the preservation of biological activity.

### 4.6. Pre-Clinical Safety Test

Weight variability was primarily associated with sex, with females presenting lower body weights, as described in the methodology. From a statistical perspective, sex was the main source of variability, accounting for most of the dispersion observed in the dataset. Importantly, no systematic differences were identified among formulations, indicating that weight changes were not associated with hydrogel administration. Furthermore, as no variations exceeding the established physiological range were observed and no abnormal behavioral signs were detected, these findings support the safety of the EO-loaded hydrogels.

Consistent with these results, histological evaluation across experimental groups revealed no appreciable morphological differences in epidermal or dermal organization. Skin architecture, layer thickness, and dermal connective tissue structure remained comparable among groups, indicating preservation of normal tissue integrity following treatment.

These findings are consistent with previous reports. In this context, Karami et al. evaluated a Carbopol^®^ hydrogel for the cutaneous delivery of α-bisabolol in BALB/c mice and reported histological findings in the blank and control groups comparable to those observed in this study for HCA, HLP, and HVE, thereby reinforcing the absence of treatment-related alterations and supporting the safety profile of the formulations [58]. Importantly, this study provides the first report of hydrogel formulations based on EOs from *C. alba* and *L. philippiana*, contributing to the valorization of Chilean endemic flora in biomedical applications. From a translational perspective, the results support the potential of the *L. philippiana* hydrogel as a promising candidate for further evaluation in canine patients.

The preclinical dermal safety evaluation in BALB/c mice further supported the tolerability of the formulations. Repeated topical application did not induce systemic adverse effects, behavioral alterations, erythema, edema, or histological evidence of inflammation or tissue damage. This is relevant because EOs may exert biological effects in mammalian cells depending on compound type and concentration; therefore, safety evaluation is necessary when developing EO-based topical products [41]. Nevertheless, safety in mice should be interpreted as a preliminary preclinical step and does not replace safety evaluation in canine patients under clinical conditions.

### 4.7. Microbiological Evaluation in Dogs

The preliminary microbiological evaluation in dogs provides relevant in vivo information regarding the potential antimicrobial performance of the formulations in the target species. Two complementary outcomes were evaluated: bacterial presence/absence and bacterial load. At baseline, all dogs were positive for *Staphylococcus* spp., confirming the infectious nature of the lesions. This is consistent with the central role of *S. pseudintermedius* as the main bacterial agent associated with canine superficial pyoderma [2,28]. On day 21, bacterial elimination was observed in the active treatment groups, whereas no elimination was detected in the vehicle group. Although elimination rates were moderate and differences were not statistically significant, this pattern suggests that complete bacterial clearance was associated with the presence of active compounds rather than with the hydrogel base alone.

In addition to bacterial elimination, the analysis of bacterial load provided a more nuanced interpretation of the microbiological response. All dogs started with very high bacterial counts at baseline, and on day 21, a shift toward lower bacterial burden categories was observed in the active treatment groups. The HLP group showed the most favorable profile, with the highest proportion of dogs reaching the non-significant bacterial load category and the lowest proportion remaining in the very high category. In contrast, no dog in the vehicle group reached the non-significant category, and all dogs remained culture-positive. These descriptive observations indicate that bacterial load may be a useful exploratory endpoint for future adequately powered studies, but no definitive conclusions regarding treatment efficacy can be drawn from the present dataset.

From a clinical perspective, this distinction is relevant. In canine superficial pyoderma, the therapeutic goal is not only bacterial eradication but also reduction in bacterial burden, control of lesion progression, and support of skin barrier recovery. Current guidelines emphasize appropriate diagnosis, cytology, bacterial culture when indicated, topical therapy for surface and superficial pyodermas, and antimicrobial stewardship to reduce unnecessary systemic antimicrobial use [28,29]. In this context, a topical formulation capable of reducing bacterial burden may have clinical value as part of a broader therapeutic strategy, even if complete microbiological clearance is not achieved in all cases.

The difference between moderate in vitro activity and the observed in vivo microbiological trends also highlights the complexity of translating antimicrobial assays into clinical outcomes. In vivo bacterial dynamics are influenced by lesion type, skin barrier status, host immune response, grooming behavior, environmental exposure, and adherence to topical application. These factors are particularly relevant in canine pyoderma, which is often associated with underlying barrier disruption, inflammation, or recurrent dermatological disease [2,28]. Therefore, the dog study should be interpreted as exploratory and hypothesis-generating rather than confirmatory. The absence of statistical significance is likely related to the limited sample size, heterogeneity of clinical cases, and uneven group sizes.

The findings should also be considered in the broader context of antimicrobial resistance. *S. pseudintermedius* is a major canine pathogen, and MRS represent an important therapeutic challenge in veterinary medicine [2,59]. Current antimicrobial use recommendations increasingly prioritize topical approaches when appropriate, especially for surface and superficial pyodermas, reserving systemic antimicrobials for cases where topical therapy is insufficient or when deeper infection is present [29]. Therefore, the development of safe topical formulations with antimicrobial potential remains clinically relevant.

This study has several limitations. First, the antibacterial activity of the final hydrogel formulations was not assessed through standardized in vitro assays such as hydrogel diffusion testing, release-medium MIC, time-kill assays, or antibiofilm assays. These studies would provide mechanistic information regarding compound release and direct antimicrobial activity of the final formulation. This is particularly relevant because topical antimicrobial performance depends not only on the intrinsic activity of the active compound but also on release kinetics, formulation stability, local retention, and interaction with the target tissue [60]. Second, the in vivo evaluation was preliminary and not powered to demonstrate treatment efficacy. Third, clinical lesion scores were not systematically analyzed alongside microbiological outcomes. Finally, the contribution of individual essential oil compounds and their possible synergistic interactions was not experimentally determined.

Future studies should therefore include direct antimicrobial testing of the final hydrogel formulations, release and stability studies of the active compounds, biofilm models involving *S. pseudintermedius*, and controlled clinical trials with larger sample sizes and standardized clinical scoring systems. These studies will be necessary to determine whether the microbiological trends observed here translate into clinically meaningful improvement in dogs with pyoderma. This is particularly important because current recommendations for canine pyoderma increasingly emphasize evidence-based selection of topical therapies and responsible antimicrobial use [29].

Overall, while the intrinsic antibacterial activity of the EOs was moderate, their incorporation into physicochemically stable, safe and microbiologically effective hydrogel formulations represents a relevant step toward the development of plant-derived topical candidates for veterinary dermatology. Among the formulations evaluated, HLP showed the most favorable overall profile, demonstrating advantageous properties in stability testing, dermal safety evaluation, and preliminary microbiological assessment, thereby supporting its selection as the lead prototype for further development. These findings support further investigation of *L. philippiana* EO-loaded hydrogels as topical candidates for canine pyoderma, while avoiding overinterpretation of the current exploratory data.

## 5. Conclusions

This study evaluated hydrogel-based delivery systems incorporating EOs from the Chilean endemic species *C. alba* and *L. philippiana* as potential topical approaches for the management of canine pyoderma. The work integrates phytochemical characterization, in vitro antibacterial activity, formulation development, microbiological quality control, stability assessment, preclinical dermal safety, and a preliminary in vivo microbiological evaluation in dogs.

Both EOs exhibited antibacterial activity against *S. pseudintermedius* clinical isolates, with chemical profiles dominated by sabinene and eucalyptol in CA-EO, and linalool, eucalyptol, and safrole in LP-EO. Although the antibacterial activity was moderate, these findings are consistent with previous reports for EOs with similar compositions, and variability among strains is expected.

All hydrogel formulations were microbiologically safe and showed physicochemical characteristics compatible with topical application on canine skin. Preclinical evaluation in mice confirmed a favorable dermal safety profile, with no evidence of irritation, systemic toxicity, or histological alterations following repeated application.

Importantly, this study provides the first report of hydrogel formulations incorporating EOs from these Chilean endemic species, contributing to the valorization of native plant resources in veterinary medicine.

A preliminary in vivo microbiological evaluation in dogs with naturally occurring pyoderma showed that EO-loaded hydrogels showed numerical differences in bacterial detection and bacterial load categories compared with the vehicle group, but these differences were not statistically significant and therefore should be interpreted cautiously. Although these differences did not reach statistical significance, a consistent trend towards lower bacterial burden particularly for HLP suggests a potential biological effect under clinically relevant conditions.

Overall, these findings support the feasibility and safety of EO-loaded hydrogels and indicate their potential as adjunct topical approaches for the management of canine pyoderma. Further studies are required to confirm clinical efficacy, including controlled trials in canine patients with larger and statistically robust sample sizes, as well as characterization of drug release, skin penetration, and pharmacodynamic behavior.

## 6. Patents

Bacterial Hydrogel INAPI national registration 202102803 10.21.2025. Bacterial Hydrogel international registration PCT/CL2020/05007 01.20.2022.

## Figures and Tables

**Table 2:** Score criteria for measuring erythema and edema in the irritation test.

Score	Erythema	Edema
0	Without lesion	Without edema
1	Slightly reddened	Slight edema
2	Moderately reddened	Moderately sized edema (>1 mm elevation)
3	Severely reddened or burnt	>1 mm elevation and extended beyond treated area

**Table 3:** Chemical composition of the CA-EO and LP-EO determined by GC-MS expressed as % of the total chromatogram area.

Retention Time (min)	IK exp	CAS #	Compound Name	% CA-EO	% LP-EO
16.1	929.6	28634-89-1	beta-Thujene	0.71	0.18
16.36	937.1	80-56-8	α-Pinene	4.38	2.01
16.93	953.1	79-92-5	Camphene	0.57	-
17.81	976.7	3387-41-5	Sabinene	13.95	2.68
17.96	980.7	18172-67-3	β-Pinene	5.72	3.67
18.36	990.9	123-35-3	β-Myrcene	1.25	0.59
18.92	1006.5	99-83-2	α-Phellandrene	0.26	2.97
19.11	1012.6	-	unknown	0.38	-
19.32	1019.2	99-86-5	α-Terpinolene	2.36	0.20
19.6	1028.0	99-87-6	p-Cymene	2.51	0.62
19.74	1032.3	5989-27-5	D-Limonene	2.70	1.26
19.86	1036.0	470-82-6	Eucalyptol	12.05	24.54
20.31	1049.7	13877-91-3	β-Ocimene	2.85	-
20.37	1051.5	13466-78-9	3-Carene	0.17	0.17
20.74	1062.4	99-85-4	γ-Terpinene	4.09	0.27
21.75	1091.4	586-62-9	α-Terpinolene	0.95	0.33
21.85	1094.2	-	unknown	-	0.22
22.04	1099.4	78-70-6	Linalool	0.96	37.91
22.4	1111.7	84254-81-9	Butanoic acid, 2-methyl-, 3-methyl-3-butenyl ester	2.18	-
22.5	1115.1	54410-94-5	Butanoic acid, 3-methyl-, 3-methyl-3-butenyl ester	1.02	-
22.99	1131.5	7216-56-0	Neo-allo-ocimene	0.26	-
24.64	1184.4	562-74-3	Terpinen-4-ol	7.03	0.56
25.06	1197.3	10482-56-1	L-α-Terpineol	0.28	4.55
26.88	1261.2	-	unknown	0.31	-
27.93	1296.4	94-59-7	Safrole	-	11.42
29.24	1345.6	20307-84-0	δ-Elemene	1.14	-
29.58	1358.2	17699-14-8	α-Cubebene	0.64	-
30.01	1373.9	97-53-0	Eugenol	-	0.33
30.38	1387.2	3856-25-5	Copaene	0.67	-
30.73	1399.6	515-13-9	β-Elemene	1.50	-
30.88	1405.7	93-15-2	Methyleugenol	-	4.29
31.3	1422.8	18252-46-5	cis-α-Bergamotene	0.15	-
31.5	1430.9	-	unknown	0.24	-
31.62	1435.7	87-44-5	Caryophyllene	1.20	0.49
31.82	1443.7	13474-59-4	trans-α-Bergamotene	2.91	-
31.94	1448.4	-	unknown	0.20	-
32.12	1455.5	-	unknown	-	0.13
32.12	1455.5	36577-33-0	Guaia-6,9-diene	3.44	-
32.38	1465.7	267665-20-3	Cadina-3,5-diene	0.41	-
32.5	1470.4	6753-98-6	Humulene	1.09	-
32.78	1481.2	-	unknown	-	0.14
32.91	1486.2	-	unknown	0.41	-
33.17	1496.2	23986-74-5	Germacrene D	2.23	-
33.18	1496.6	-	unknown	-	0.21
33.26	1499.6	-	unknown	0.39	-
33.31	1501.7	-	unknown	-	0.13
33.35	1503.4	-	unknown	0.83	-
33.56	1512.4	24703-35-3	Bicyclogermacrene	1.26	-
33.74	1520.1	-	unknown	0.63	-
33.75	1520.5	-	unknown	-	0.12
34.15	1537.3	483-77-2	Calamenene	3.24	-
34.44	1549.4	-	Furopelargone A Isomer	1.20	-
34.69	1559.8	21391-99-1	α-Calacorene	0.22	-
34.87	1567.2	40716-66-3	trans-Nerolidol	1.68	-
35.66	1599.2	1143-45-9	Furopelargone A	4.65	-
35.77	1604.1	1139-30-6	Caryophyllene oxide	0.59	-
36.03	1615.8	-	unknown	0.35	-
36.38	1631.5	19888-34-7	Humulene epoxide II	0.44	-
36.72	1646.6	21284-22-0	Cubenol	0.40	-
37.05	1661.1	-	unknown	0.26	-
37.36	1674.6	473-15-4	Eudesmol	0.58	-
45.67	2083.7	562-28-7	Kaur-16-ene	0.27	-

**Table 4:** Average inhibition zone diameter (mm) of CA-EO and LP-EO against *S. pseudintermedius* (SP) clinical isolates. Values represent the mean ± standard deviation of inhibition zone diameters obtained from three independent replicates.

Strain	CC	CA-EO	LP-EO	CHX (2%)
SP 1	+	8.28 ± 0.43	8.05 ± 0.79	24.39 ± 0.97
SP 3	+	8.20 ± 0.30	6.62 ± 0.31	25.46 ± 1.44
SP 6	+	8.40 ± 0.43	7.72 ± 0.71	23.32 ± 0.34
SP 7	+	7.56 ± 0.73	8.62 ± 0.69	24.08 ± 1.11
SP 8	+	7.39 ± 0.32	4.92 ± 0.26	22.85 ± 0.61
SP 9	+	10.38 ± 0.09	6.68 ± 0.37	27.21 ±1.10
SP 10	+	7.99 ± 0.33	9.89 ± 0.51	23.26 ±0.47
SP 11	+	8.43 ± 0.40	7.29 ± 0.21	20.41 ± 0.94
SP 12	+	9.11 ± 0.52	6.32 ± 0.09	24.02 ± 0.46
SP 13	+	8.59 ± 0.64	10.80 ± 0.20	26.04 ± 0.51
SP 14	+	8.00 ± 0.30	7.06 ± 0.31	24.09 ± 0.83
SP ATCC 49444	+	9.63 ± 0.66	8.89 ± 0.09	24.08 ± 0.45

Chlorhexidine 2% (CHX) was used as a reference antiseptic control. CC: culture control (growth without treatment). SP ATCC 49444 corresponds to the reference strain.

**Table 5:** Minimum inhibitory concentrations (MICs) of CA-EO and LP-EO against *S. pseudintermedius* (SP) clinical isolates. MIC values are expressed in mg mL^−1^. The reference antiseptic used was chlorhexidine (CHX) 2%. SP ATCC 49444 corresponds to the reference strain. DMSO 5%.

Strain	CA-EO	LP-EO	CHX (2%)
SP 1	>4.096	4.096	0.128
SP 3	>4.096	4.096	0.064
SP 6	>4.096	4.096	0.064
SP 7	>4.096	4.096	0.128
SP 8	4.096	4.096	0.064
SP 9	4.096	4.096	0.128
SP 10	>4.096	4.096	0.128
SP 11	>4.096	>4.096	0.128
SP 12	>4.096	2.048	0.128
SP 13	>4.096	4.096	0.128
SP 14	>4.096	4.096	0.128
SP ATCC 49444	>4.096	4.096	0.064

**Table 6:** Bacterial strains identified by VITEK^®^ between day 1 and day 21 in dogs with superficial pyoderma (*n = 32*). The data represents the number of dogs showing each transition in bacterial species between baseline (day 1) and at the end of treatment (day 21). “Other spp.” includes *Staphylococcus capitis* and *Staphylococcus warneri*. “No bacteria” indicates absence of detectable cocci in culture on day 21.

		Strain Identified on Day 21			
		*S. pseudintermedius*	*S. aureus*	Other spp.	No Bacteria
Strain	*S. pseudintermedius*	15	4	1	7
	*S. aureus*	2	2	0	1
	Other spp.	1	0	0	0

**Table 7:** Bacterial presence and absence on day 21 by treatment group in dogs with superficial pyoderma. Presence indicates detection of any *Staphylococcus* species by culture and VITEK^®^ identification on day 21. Absence indicates no detectable cocci in culture. HCA: hydrogel containing *Cryptocarya alba* essential oil; HLP: hydrogel containing *Laureliopsis philippiana* essential oil; HVE: vehicle hydrogel.

Group	n	Presence	Absence
HCA	8	6	2 (25.0%)
HLP	8	6	2 (25.0%)
HVE^®^	6	6	0 (0.0%)
Inveclor	8	4	4 (50%)

**Table 8:** Bacterial load categorization on day 21 by treatment group. Bacterial load was categorized based on CFU counts on day 21. Non-significant: 0–15 CFU/swab; Countable: 15–300 CFU/swab; Uncountable: >300 CFU/swab.

Group	n	Non-Significant	Countable	Uncountable
HCA	8	3 (37.5%)	2 (25.0%)	3 (37.5%)
HLP	8	6 (75%)	1 (12.5%)	1 (12.5%)
HVE	6	0 (0%)	4 (66.7%)	2 (33.3%)
Inveclor^®^	8	4 (50%)	1 (12.5)	3 (37.5%)

## Data Availability

The original contributions presented in this study are included in the article/Supplementary Materials. Further inquiries can be directed to the corresponding author.

## Supplementary Materials

The following supporting information can be downloaded at https://www.mdpi.com/article/10.3390/vetsci13060544/s1: S1: Table S1: Density and pH of the hydrogel formulations HCA, HLP, and HVE. S2: Figure S1: Rheological characterization of the HCA, HLP, and HVE formulations. S3: Figure S2: Microbiological safety (quality control) assay to assess pathogen growth following inoculation of 10 µL of hydrogel samples (a) HCA, (b) HLP, and (c) HVE on blood agar and Mueller–Hinton agar, followed by incubation for 24 h at 36.5 °C. S4: Figure S3: Macroscopic appearance of HCA, HLP, and HVE formulations during the thermal stability assay at 50 °C. S5: Table S2: Number of phases and odor of HLP, HCA, and HVE during the thermal stability test at 50 °C. S6: Table S3: Initial (0 h) and final (120 h) pH values for HCA, HLP, and HVE during the thermal stability test. The thermal stability test was carried out 2 weeks after formulation. pH measurements were taken once each formulation reached 20–25 °C. S7: Figure S4: Macroscopic appearance of 1 mL hydrogel samples before and after centrifugation at 13,000 RPM for 10 min. S8: Table S4: Number of phases, odor score, and viscosity score of HCA, HLP, and HVE after 6 months of storage (long-term stability test).

## Abbreviations

The following abbreviations are used in this manuscript:

CA	*Cryptocarya alba*
CA-EO	*Cryptocarya alba* essential oil
CHX	Chlorhexidine
EO	Essential oil
HCA	Hydrogel carrying CA-EO
HLP	Hydrogel carrying LP-EO
HVE	Hydrogel vehicle
LP	*Laureliopsis. philippiana*
LP-EO	*Laureliopsis philippiana* essential oil
MIC	Minimum inhibitory concentration
MHB	Mueller–Hinton Broth
MRS	Meticillin-resistant strains
SP	*Staphylococcus pseudintermedius*
