# Supplementary material for: Cryptocarya alba and Laureliopsis philippiana Essential Oil-Loaded Hydrogels with Antibacterial Activity Against Staphylococcus pseudintermedius: Potential Topical Candidates for Canine Pyoderma
Source: Vet Sci. 2026 May 31;13(6):544. doi: 10.3390/vetsci13060544 (PMC13307746; doi:10.3390/vetsci13060544)
Supplement: Supplementary file 1 [file vetsci-13-00544-s001.zip › vetsci-4274023-supplementary.pdf]

veterinary sciences

MDPI

Supplementary material

Cryptocarya alba and *Laureliopsis philippiana* Essential Oil-Loaded Hydrogels with Antibacterial Activity Against *Staphylococcus pseudintermedius*: Potential Topical Candidates for Canine Pyoderma

Martina Jacobs [1,2], Noelia Valdivia [1], Martín Varas [1,2], Paola Ramos [1], Flavia Bruna [1,3,4], Gabriela Valenzuela [1,5], Olosmira Correa [1,5], Antonia Díaz [1,6], Gabriela Maturana [1], Irene Martínez [2], Francisco Abusleme [7,8], Belén Rivera [8], María Olga Bargsted [7], Daniela Siel [7,9,*] and Jessica Bravo [1,*]

[1] Laboratory of Bioactive Natural Products, School of Medicine, Center for Biomedical Research, University Diego Portales, Ejército 141, Santiago 8370007, Chile; martinajacobs@ug.uchile.cl (M.J.); noelia.valdivia_c@mail.udp.cl (N.V.); martin.varas@ug.uchile.cl (M.V.); proyectos.pramos@gmail.com (P.R.); flabruna@gmail.com (F.B.); gabriela.m.valenzuela@ciq.uchile.cl (G.V.); ocorrea@ciq.uchile.cl (O.C.); antonia.diazp@mayor.cl (A.D.); gabriela.maturana.alvarez@gmail.com (G.M.)
[2] Department of Chemical Engineering, Biotechnology and Materials, Faculty of Physical and Mathematical Sciences, University of Chile, Beauchef 851, Santiago 8370458, Chile; imartinez@ing.uchile.cl
[3] Hormone and Cancer Biology Laboratory, Institute of Experimental Medicine and Biology of Cuyo (IMBECU), CONICET CCT Mendoza—National University of Cuyo (UNCuyo), Mendoza 5500, Argentina
[4] Center of Odontological Research (CIO) from the Faculty of Odontology, National University of Cuyo (UNCuyo), Mendoza 5500, Argentina
[5] Department of Pharmacological Chemistry and Toxicology, Faculty of Chemical and Pharmaceutical Sciences, University of Chile, Carlos Lorca Tobar 964, Santiago 8380492, Chile
[6] School of Biotechnology, Faculty of Sciences, Engineering and Technology, Mayor University, Camino La Pirámide 5750, Santiago 8580000, Chile
[7] School of Veterinary Medicine, Faculty of Medicine and Health Sciences, Mayor University, Camino La Pirámide 5750, Santiago 8580000, Chile; fabusleme@gmail.com (F.A.); mobargsted@gmail.com (M.O.B.)
[8] Oftaderm Veterinary Clinic, Av. Francisco Bilbao 7341, La Reina, Santiago 7850003, Chile; belen.rivera@oftaderm.cl
[9] Center for Biomedicine, Mayor University, Camino La Pirámide 5750, Santiago 8580000, Chile
* Correspondence: daniela.siel@umayor.cl (D.S.); jessica.bravo@mail.udp.cl (J.B.)

1. Supplementary material S1

Table S1. Density and pH of the hydrogel formulations HCA, HLP, and HVE.

Formulation	Density	pH
HCA	1.02±0.02 [a]	7.68±0.03 [b]
HLP	1.05±0.04 [a]	7.74±0.02 [c]
HVE	1.04±0.02 [a]	7.72±0.02 [b, c]

Different letters indicate statistically different values ($p < 0.05$).

2. Supplementary material S2

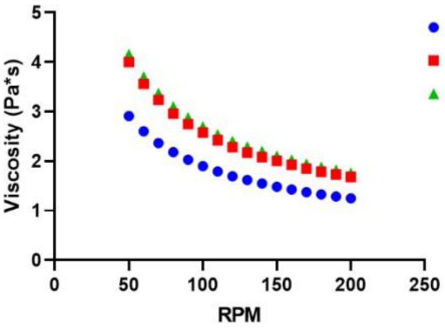

(a)

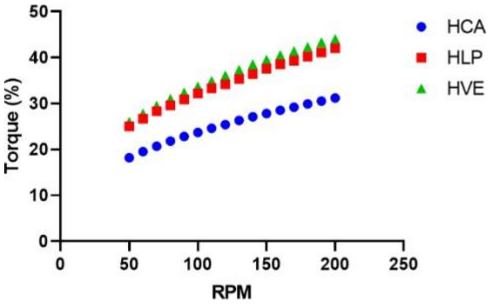

(b)

Figure S1. Rheological characterization of the HCA, HLP, and HVE formulations. (a) Viscosity and (b) Torque.

3. Supplementary material S3

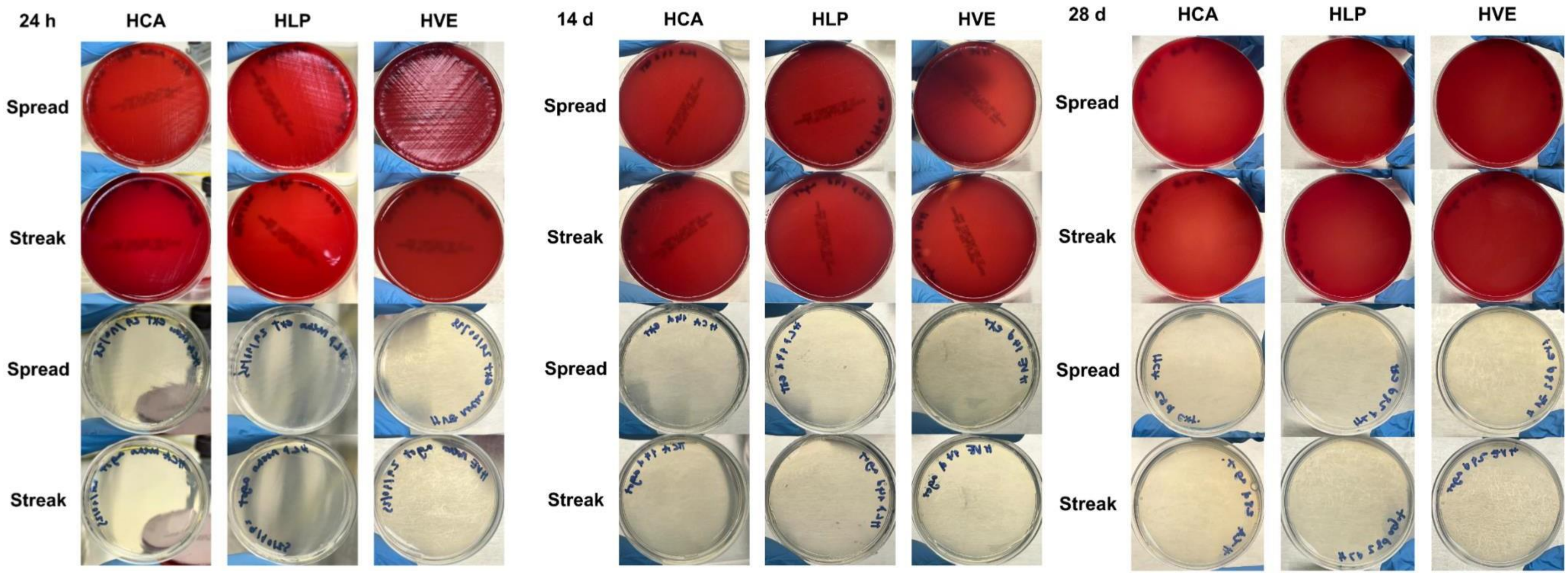

(**a**) (**b**) (c)

Figure S2. Microbiological safety (quality control) assay to assess pathogen growth following inoculation of 10 μL of hydrogel samples (a) HCA, (b) HLP, and (c) HVE on blood agar and Mueller–Hinton agar, followed by incubation for 24 hours at 36.5 °C.

4. Supplementary material S4

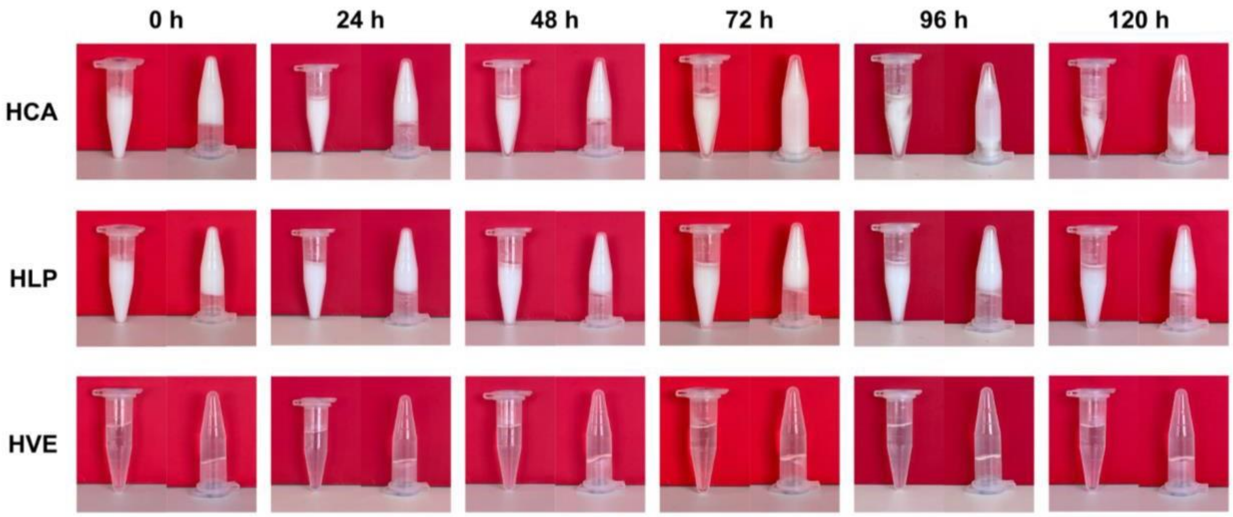

Figure S3. Macroscopic appearance of H-CA, H-LP, and H-VE formulations during the thermal stability assay at 50 °C.

5. Supplementary material S5

Table S2. Number of phases and odor of HLP, HCA, and HVE during the thermal stability test at 50°C.

Time	HCA		HLP		HVE	
(h)	# Phases	Odor	# Phases	Odor	# Phases	Odor
0	1	Characteristic CA odor. Score 4.	1	Characteristic LP odor. Score 4.	1	Odorless. Score 0.
24	3	Characteristic CA odor. Score 4.	2	Characteristic LP odor. Score 3.	1	Odorless. Score 0.
48	3	Characteristic CA odor. Score 4.	2	Characteristic LP odor. Score 3.	1	Odorless. Score 0.
72	3	Characteristic CA odor. Score 4.	3	Characteristic LP odor. Score 3.	1	Odorless. Score 0.
96	3	Characteristic CA odor. Score 3.	3	Characteristic LP odor. Score 3.	1	Odorless. Score 0.
120	3	Characteristic CA odor. Score 3.	3	Characteristic LP odor. Score 3.	1	Odorless. Score 0.

6. Supplementary material 6

Table S3. Initial (0 h) and final (120 h) pH values for HCA, HLP, and HVE during the thermal stability test. The thermal stability test was carried out 2 weeks after formulation. pH measurements were taken once each formulation reached 20-25°C.

Sample	pH (0 h)	pH (120 h)
HCA	7.75±0.04 ^{a}	7.35±0.10 ^{b}
HLP	7.81±0.05 ^{a}	7.66±0.05 ^{a}

HVE	7.70±0.04 [a]	7.72±0.04 [a]

Different letters indicate statistically different values ($p < 0.05$).

7. Supplementary material S7

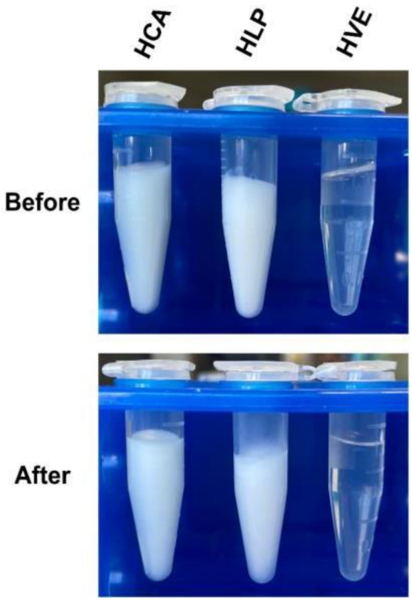

Figure S4. Macroscopic appearance of 1 mL hydrogel samples before and after centrifugation at 13,000 rpm for 10 minutes.

8. Supplementary material S8

Table S4. Number of phases, odor score, and viscosity score of HCA, HLP, and HVE after 6 months of (long-term storage stability test).

Formulation	# phases	Odor
HCA	3	Characteristic CA odor. Score 4.
HLP	2	Characteristic LP odor. Score 4.
HVE	1	Odorless. Score 0.

Abbreviations

HCA	Hydrogel carrying *Cryptocarya alba* essential oil
HLP	Hydrogel carrying *Laureliopsis philippiana* essential oil
HVE	Hydrogel vehicle
